# Polyunsaturated Fatty Acids and Reduced Risk of Low Muscle Mass in Adults

**DOI:** 10.3390/nu17050858

**Published:** 2025-02-28

**Authors:** Haiyu Zou, Liangrong Zheng, Chunlai Zeng

**Affiliations:** School of Medicine, Zhejiang University, Hangzhou 310000, China; 22218287@zju.edu.cn

**Keywords:** fatty acids, low muscle mass, WQS, mediation effect, linoleic acid, alpha-linolenic acid

## Abstract

**Background**: We aimed to evaluate the effects of both joint and individual types of fatty acids on low muscle mass in adults. **Methods**: We enrolled 8842 adults selected from the National Health and Nutrition Examination Survey (NHANES). Multivariate adjusted weighted logistic regression models were employed to evaluate the connection between fatty acids and low muscle mass. We used restricted cubic splines (RCSs) to determine whether the relationship is linear or non-linear, while stratified analyses and interaction effects were also assessed. Weighted quantile sum (WQS) analysis assessed the impact of joint and individual types of fatty acids on low muscle mass. Additionally, mediation analysis determined the direct and indirect implications of polyunsaturated fatty acids on low muscle mass. **Results**: A total of 8842 participants were included in this study, of which 705 were identified as having low muscle mass. The logistic regression analyses identified a significant linear correlation between all three types of fatty acids and low-muscle-mass risk. Additionally, the WQS analysis demonstrated that a fatty acid mixture was inversely associated with low-muscle-mass risk, with polyunsaturated fatty acids being recognized as the principal component. Moreover, inflammation may mediate the relationship between polyunsaturated fatty acids and low muscle mass, accounting for 3.75% of the effect size (*p* < 0.001) through white blood cell count. We further examined linoleic acid (LA) and alpha-linolenic acid (ALA), and each unit increase in LA and ALA intake was linked to a decrease in low-muscle-mass risk by 0.29 (95% CI: 0.64–0.79, *p* < 0.001) and 0.27 (95% CI: 0.66–0.81, *p* < 0.001), respectively. **Conclusions**: These findings indicate that polyunsaturated fatty acids (especially LA and ALA) may effectively mitigate low-muscle-mass risk.

## 1. Introduction

Given the rapid growth of the global aging population and the inevitable regression in muscle mass and function with age, addressing skeletal muscle health has become increasingly essential [1]. Sarcopenia, in particular, characterized by a progressive and age-related decline in muscle mass and function, constitutes a severe threat to the well-being and independence of elderly individuals [2]. However, low muscle mass can occur at any age and in the context of chronic or acute conditions [3]. The impact of low muscle mass extends beyond sarcopenia, as skeletal muscle fulfills various functional and metabolic roles [4]. Recognizing its clinical importance, the Global Leadership Initiative on Malnutrition (GLIM) has identified low muscle mass as a key diagnostic criterion for malnutrition [5]. Therefore, studying low muscle mass across a diverse adult population—encompassing both aging individuals and those affected by metabolic conditions or other health factors—is essential. Currently, the Food and Drug Administration has not approved any medications specifically for low muscle mass. Due to the lack of an effective cure, understanding the factors contributing to low muscle mass in adults of all ages is crucial, as early intervention may help prevent or slow its progression.

Dietary factors have garnered increasing attention in the progression of low muscle mass. Research has demonstrated that nutritional factors are crucial in managing low muscle mass [6]. Evidence suggests that fatty acids can significantly enhance muscle metabolism [7]. Fatty acids are integral to various physiological processes, including inflammation [8] and muscle lipid metabolism [9], which are crucial to sustaining muscle health. In adults, shifts in fatty acid metabolism may contribute to early muscle decline, potentially setting the stage for more severe low muscle mass later in life.

Despite the increasing interest in dietary contributors to muscle mass, the specific relationship between fatty acids and low muscle mass in adults is insufficiently researched. Our study addresses this gap by exploring the association between joint and individual types of fatty acids and low muscle mass.

## 2. Materials and Methods

### 2.1. Data Sources

**Figure 1:**
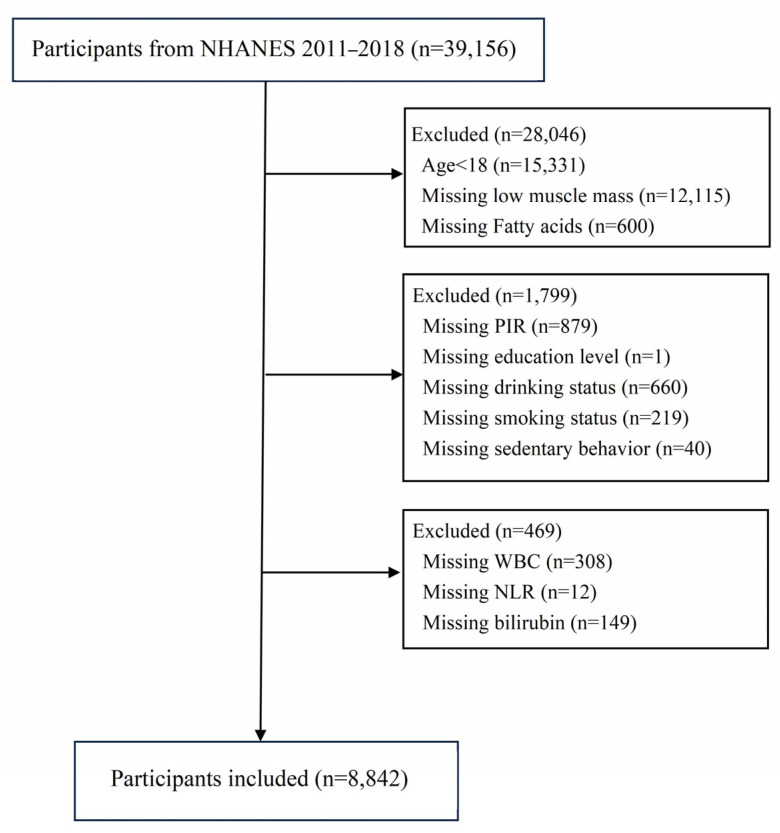
Workflow.

We analyzed data from four NHANES cycles spanning 2011–2018. We included individuals aged 18 and above who had undergone dual-energy X-ray absorptiometry (DXA) (Hologic, Inc., Bedford, MA, USA). Participants below 18 or lacking complete data on DXA, fatty acids, covariates, white blood cells, neutrophils, or bilirubin were excluded. Ultimately, we enrolled 8842 participants, as illustrated in Figure 1.

### 2.2. Definition of Low Muscle Mass

Low muscle mass was delineated using criteria created by the Foundation for the National Institutes of Health [10]. Specifically, it was defined as skeletal muscle mass adjusted for body mass index (ASMBMI) values below 0.512 kg/m^2^ for females and below 0.789 kg/m^2^ for males. The skeletal muscle mass of the arms and legs was quantified using dual-energy X-ray absorptiometry (DXA). All scans were conducted using Hologic Discovery model A densitometers (Hologic, Inc., Bedford, MA, USA) with software version Apex 3.2 (Hologic, Inc., Bedford, MA, USA). To guarantee safety, some specific populations were restricted from DXA screening, including gravid women and those with a weight exceeding 136 kg or a height surpassing 196 cm.

### 2.3. Fatty Acid Assessment

NHANES employs an automated multiple-pass method to assess and quantify individuals’ 24 h dietary intake [11]. Between 2011 and 2018, two 24 h dietary recalls were conducted in each NHANES cycle by certified interviewers proficient in Spanish and English. The first recall occurred during personal interviews in the Mobile Examination Center (MEC), where standardized protocols were consistently followed. The second recall was carried out by phone within 3 to 10 days of the first MEC assessment. Dietary fatty acid intake was evaluated by the Food and Nutrition Database for Dietary Studies [12]. Saturated, monounsaturated, and polyunsaturated fatty acids were collected. Specifically, the saturated fatty acids (SFAs) included caproic acid (4:0), caprylic acid (6:0), capric acid (8:0), lauric acid (10:0), myristic acid (12:0), palmitic acid (14:0), stearic acid (16:0), and arachidic acid (18:0). The monounsaturated fatty acids (MFAs) analyzed consisted of palmitoleic acid (16:1), oleic acid (18:1), eicosenoic acid (20:1), and erucic acid (22:1). The polyunsaturated fatty acids (PFAs) examined included linoleic acid (18:2), α-linolenic acid (18:3), stearidonic acid (18:4), arachidonic acid (20:4), eicosapentaenoic acid (EPA, 20:5), docosapentaenoic acid (DPA, 22:5), and docosahexaenoic acid (DHA, 22:6). Quartiles were utilized to stratify participants into four equal groups based on their fatty acid levels, which facilitated the analysis of potential dose–response relationships and allowed for the assessment of trends across different levels of exposure.

### 2.4. Definition of Covariates

A variety of demographic and fitness-related variables were collected during NHANES household interviews, such as age, gender, ethnicity, household economic level, educational attainment, smoking and drinking status, and medical history. The population’s ethnicity was grouped into four: Mexican Americans, Non-Hispanic White people, Non-Hispanic Black people, and other races. The household economic level was defined using the poverty income ratio (PIR) as low (less than 1.0), middle (1.0 to 3.0), and high (over 3.0). Educational attainment was divided into two categories—high school or over and below high school—with the former group including college graduates and those with some college education or a GED. Self-reported data on smoking, alcohol consumption, diabetes mellitus, and cardiovascular disease were also analyzed. Smoking status was binary, with “no” indicating individuals who never smoked and “yes” for those who had smoked or were currently smoking. Excessive alcohol consumption was identified through a dummy variable indicating current heavy or moderate drinking. Sedentary behavior was evaluated using an authenticated, self-completed physical activity questionnaire, which was tested to be reliable [13]. Patients with cardiovascular disease were identified based on a history of myocardial infarction, congestive heart failure, angina, stroke, or heart attack. The diagnostic criteria for diabetes mellitus encompass elevated glycohemoglobin levels (>6.5%), random blood glucose measurements (≥11.1 mmol/L), fasting blood glucose levels (≥7.0 mmol/L), physician confirmation, glucose level from a two-hour oral glucose tolerance test over 11.1 mmol/L, or current administration of diabetic medications or insulin. Bilirubin and gamma-glutamyl transferase were selected to indicate oxidative stress [14,15]. Furthermore, white blood cell counts were utilized as indicators to evaluate chronic inflammation.

### 2.5. Statistical Analysis

Descriptive analysis was performed to compare the baseline characteristics between the low-muscle-mass group and the non-low-muscle-mass group. The frequency proportions showed categorical variables, whereas the mean and the first and third quartiles showed continuous variables. The normality of continuous variables was assessed using the Shapiro–Wilk test. Multivariable logistic regression evaluated the association between three types of fatty acids and low-muscle-mass prevalence. The crude model adjusted nothing. Model 1 was adjusted for age, gender, ethnicity, household economic level, and educational attainment. Model 2 was further adjusted for smoking status, alcohol use, sedentary behavior, diabetes mellitus, hypertension, and obesity based on model 1. A linear trend analysis was performed across the fatty acid quartiles, with the first quartile as the reference. Additionally, WQS analysis was employed to evaluate the joint and individual effects of three types of fatty acids on low-muscle-mass risk by generating a weighted linear index. Sensitivity analyses and subgroup interactions were explored by stratifying the population based on age, gender, ethnicity, household economic level, educational attainment, smoking status, alcohol consumption, sedentary lifestyle, diabetes, hypertension, and obesity. Mediation analysis, conducting nonparametric bootstrapping (*n* = 1000), was applied to investigate immediate and mediated effects and the magnitude of mediating pathways. R software (version 4.4.2, R Foundation for Statistical Computing, Vienna, Austria) was used for all statistical analyses, and a significance level of *p* < 0.05 was set.

## 3. Results

### 3.1. Baseline Characteristics of Study Participants

Table 1 delineates the demographic characteristics of the 8842 participants, of whom 707 individuals (8.0%) were identified as having low muscle mass. Notable disparities were observed between the low-muscle-mass and non-low-muscle-mass groups concerning baseline variables, including age, ethnicity, household economic status, educational level, quartiles of polyunsaturated, monounsaturated, and saturated fatty acids, drinking status, obesity, sedentary behavior, diabetes, and hypertension (all *p* < 0.01). No notable differences were observed regarding gender (*p* = 0.737) or smoking status (*p* = 0.252).

### 3.2. Fatty Acids and Low-Muscle-Mass Risk in the Logistic Regression Model

Univariate and multivariate regression analyses assessed the relationship between fatty acid quartiles and low-muscle-mass risk. As shown in Table 2, a significant linear trend was observed for the fatty acids analyzed across all three models. After adjusting for covariates, including age, gender, ethnicity, household economic level, educational attainment, smoking status, drinking status, diabetes, hypertension, and obesity, the odds ratios (ORs) and 95% confidence intervals (CIs) for low muscle mass across the PUFA quartiles were [1.00 (reference)], [0.78 (0.58–1.06)], [0.71 (0.54–0.93)], and [0.48 (0.34–0.69)]. For the SFA quartiles, the ORs and 95% CIs were [1.00 (reference)], [0.71 (0.54–0.94)], [0.65 (0.47–0.89)], and [0.51 (0.35–0.76)]. Similarly, for the MUFA quartiles, the ORs and 95% CIs were [1.00 (reference)], [0.90 (0.69–1.17)], [0.64 (0.47–0.88)], and [0.52 (0.38–0.71)]. The *p*-values for the trends were 0.001 for PUFAs, 0.002 for SFAs, and 0.001 for MUFAs, respectively. The observed dose–response relationship shows the odds of having low muscle mass decreasing progressively across the fatty acid quartiles. For example, after adjusting for key demographic and clinical factors, individuals in the highest PUFA quartile had a 52% lower risk of low muscle mass compared to those in the lowest quartile. Similarly, those in the highest MUFA and SFA quartiles experienced a 48% and 49% reduction in risk, respectively.

### 3.3. The Detection of Linear Relationships Between Fatty Acid Intake and Low-Muscle-Mass Risk

**Figure 2:**
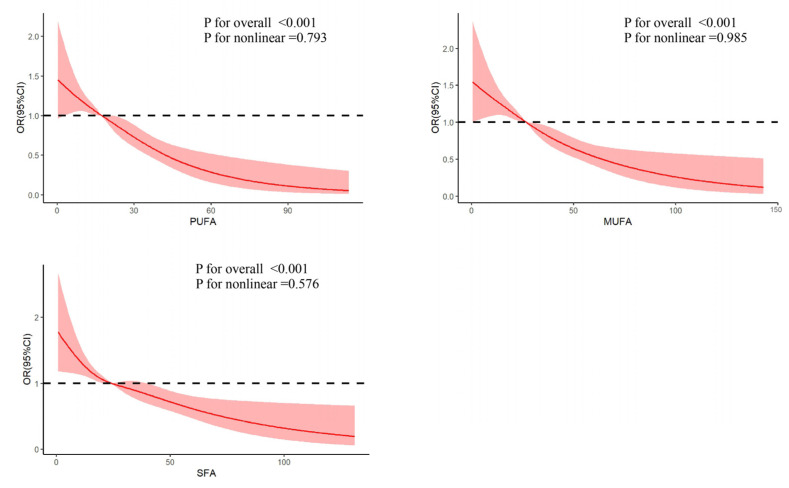
RCS analysis between fatty acids and sarcopenia.

A precise dose–response association between three types of fatty acids and low muscle mass was further identified. As depicted in Figure 2, after adjusting for all covariates, a linear association between these variables and low muscle mass was observed, with a non-linear *p*-value of 0.793, 0.985, and 0.576 for PUFAs, MUFAs, and SFAs, respectively. These findings suggest that increased dietary intake of PUFAs, MUFAs, and SFAs is related to lower low-muscle-mass risk.

### 3.4. Fatty Acid Concentrations and Low-Muscle-Mass Risk in the WQS Model

**Figure 3:**
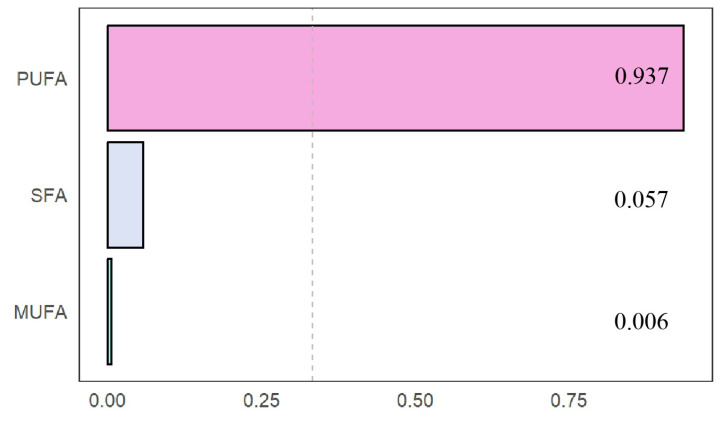
The WQS model weights of fatty acids on the prevalence of sarcopenia.

WQS was assessed to determine the combined effects of three types of fatty acids on low-muscle-mass prevalence. As shown in Table 3, WQS regression analysis revealed that for each one-unit increase in the WQS score, the log odds of low muscle mass decreased by 0.21840 (standard error = 0.04636). When converted into an odds ratio (OR), this corresponds to 10^−0.2184^, which is approximately 0.803, indicating a 19.7% reduction in the odds of low muscle mass with each unit increase in the WQS score. Clinically, this suggests that higher cumulative levels of these fatty acids are associated with a reduced risk of low muscle mass (*p* < 0.001). Figure 3 highlights that PUFAs contributed the most to low-muscle-mass risk reduction, with a weight of 0.94, compared to 0.06 for saturated fatty acids and 0.01 for monounsaturated fatty acids.

### 3.5. Subgroup Analysis

**Figure 4:**
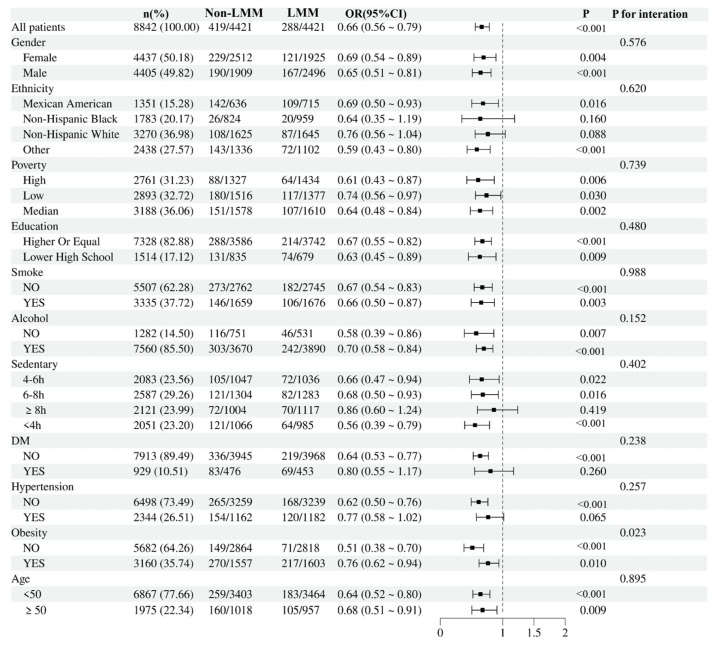
Subgroup analysis. LMM: low muscle mass.

We conducted subgroup analyses based on age, gender, ethnicity, household economic level, educational attainment, smoking status, drinking status, sedentary behavior, diabetes, hypertension, and obesity. As shown in Figure 4, the positive association between PUFAs and low-muscle-mass prevalence was consistent across most subgroups (*p* for interaction > 0.05). However, obesity was identified as a possible effect modifier, with an evident interaction between PUFAs and low muscle mass (*p* for interaction = 0.023). This suggests that obesity may play a role in how PUFAs impact the risk or presence of low muscle mass, highlighting the potential need for supplementing PUFAs in obese individuals to manage or prevent low muscle mass.

### 3.6. Mediation Effect

Table 4 illustrates the mediation analysis results, indicating that albumin, white blood cells, bilirubin, and gamma-glutamyl transferase partially mediate the relationship between PUFAs intake and low muscle mass. White blood cells had the strongest mediation effect (3.75%, *p* < 0.001), suggesting that inflammation may be a pathway linking PUFAs to low muscle mass. Albumin mediated 2.64% of this relationship (*p* = 0.03), suggesting a potential role of nutritional status in this association. Bilirubin (1.60%, *p* = 0.042) and GGT (1.14%, *p* = 0.004) also contributed to the mediation, possibly reflecting mechanisms related to oxidative stress and liver function. Despite statistical significance, the mediation proportions are small, indicating that other unexamined pathways may play a larger role.

### 3.7. Associations Between Types of Polyunsaturated Fatty Acids and Low Muscle Mass

Building on the WQS model findings, we performed a weighted logistic regression to investigate the associations between types of polyunsaturated fatty acids and low muscle mass. As depicted in Table 5, LA and ALA were remarkably linked to a reduced low-muscle-mass risk in the fully adjusted model. Precisely, each unit increase in LA and ALA corresponded to a decrease in low-muscle-mass risk of 0.29 (95% CI: 0.64–0.79, *p* < 0.001) and 0.27 (95% CI: 0.66–0.81, *p* < 0.001), respectively. All these findings suggest that increasing the intake of LA and ALA through dietary sources could help reduce the risk of low muscle mass, particularly in individuals at risk, offering a potential strategy for the prevention or management of muscle loss.

## 4. Discussion

We revealed the correlation between dietary fatty acids and low muscle mass in adults utilizing data from NHANES. We employed multivariable logistic regression analysis, WQS analysis, RCS analysis, subgroup analysis, and mediation analysis to investigate the joint and individual effects of three types of fatty acids on low muscle mass. Our findings indicate that the overall mixture of fatty acids is inversely associated with the risk of low muscle mass, with PUFAs identified as the primary contributor. Subgroup analyses demonstrated a considerable connection between PUFAs and obesity, which suggests a more pronounced relationship between PUFAs and low muscle mass in obese patients. Therefore, the appropriate addition of polyunsaturated fatty acids in the diet of obese individuals can help reduce their low-muscle-mass risk. Additionally, noteworthy dose–response relationships were identified using RCS, where all three types of fatty acids exhibited a linear relationship with low-muscle-mass risk. We also found that LA and ALA, two nutritionally essential fatty acids that mammals cannot synthesize, were significantly associated with reduced low-muscle-mass risk.

Maintaining muscle mass is essential not only for proper physical movement but also for supporting various metabolic and homeostatic functions. Low muscle mass is a prevalent condition that can occur at any stage of life due to multiple factors, including inadequate nutrition, physical inactivity, chronic diseases, and metabolic disorders such as obesity and diabetes. It has garnered significant attention in recent years, as individuals with low muscle mass face a higher risk of adverse health outcomes, including chronic obstructive pulmonary disease [16], cancer [17], and cardiovascular disease [18]. Moreover, it imposes a significant economic burden on healthcare systems, contributing to higher hospitalization costs and post-surgical complications such as infections and prolonged ventilation [19]. Considering its clinical significance, further research is needed, particularly on its prevention and the modification of risk factors.

Physical exercise and adequate nutritional support are widely recognized as key strategies for preventing its decline [20]. In particular, nutrition is vital for preserving muscle mass and function, with higher intakes of antioxidant nutrients emerging as crucial lifelong regulators [2]. Traditional nutritional interventions have largely emphasized increasing protein intake and amino acid supplementation [21,22], but emerging evidence underscores the significant effect of fatty acids in promoting muscle health. Fatty acids, as crucial components of lipids, are essential nutrients involved in cellular and tissue metabolism and function. For example, a Canadian study found that 12-week supplementation of omega-3 fatty acids enhanced muscle mass and physical function in older women [23]. Similarly, other studies have demonstrated that n-3 polyunsaturated fatty acids (PUFAs) derived from fish oil significantly improve muscle anabolic responses and enhance physical performance [24]. While earlier research has primarily concentrated on the effects of individual types of fatty acids, it is crucial to recognize that individuals are usually exposed to multiple fatty acids simultaneously. These fatty acids can interact, leading to synergistic or antagonistic effects that may alter their impact on muscle health. For instance, diets rich in omega-6 fatty acids can diminish the anti-inflammatory benefits of omega-3 fatty acids [25], and animal studies have shown that saturated fatty acids can counteract the cytotoxic impacts of unsaturated fatty acids [26]. Thus, evaluating the combined effects of types of fatty acids, as well as isolating fatty acids, can offer more meaningful insights for clinical practice on low muscle mass.

Low muscle mass results from a complex interplay of molecular pathways that regulate muscle protein synthesis and degradation. Several key transcription factors and signaling pathways contribute to muscle atrophy by enhancing proteolytic activity and inhibiting anabolic processes. One of the primary regulators of muscle atrophy is the Forkhead box O (FoxO) family of transcription factors, which plays a crucial role in the maintenance of skeletal muscle homeostasis [27]. In addition to FoxO, the nuclear factor-kappa B (NF-κB) signaling pathway is another critical contributor to muscle atrophy. Proinflammatory cytokines, such as TNF-α and IL-6, activate NF-κB, which subsequently induces the expression of genes associated with muscle degradation [28]. PUFAs have shown potential in mitigating these processes. They enhance antioxidant defenses by increasing superoxide dismutase levels and upregulating DAF-16/FOXO transcription factors, which suppress reactive oxygen species production and oxidative stress [29]. PUFAs also inhibit the NF-κB signaling pathway, leading to reduced inflammatory markers such as IL-6 and TNF-α, thereby helping to alleviate chronic inflammation associated with low muscle mass [30]. Moreover, numerous in vitro [31,32,33] and in vivo [34,35,36,37] studies have shown that both saturated fatty acids and unsaturated fatty acids, particularly omega-3 polyunsaturated fatty acids, exert beneficial effects on muscle health. Omega-3 PUFAs, in particular, have been highlighted for their role in promoting muscle maintenance and mitigating muscle degradation, making them essential in muscle preservation and overall metabolic health.

Natural and fortified food sources are essential for ensuring adequate PUFA intake. ALA, an essential omega-3 fatty acid, is abundant in plant oils such as flaxseed, soybean, and canola oils, as well as in walnuts and chia seeds. Long-chain omega-3 fatty acids, including eicosa-pentaenoic acid (EPA) and docosa-hexaenoic acid (DHA), are primarily present in fatty fish-like salmon, mackerel, tuna, herring, and sardines. Furthermore, fortified foods, including certain brands of yogurt, eggs, and algae oils, are excellent sources of DHA and other omega-3s [38]. These natural and supplemented sources play a significant role in ensuring adequate omega-3 intake for maintaining health. The complex digestion and absorption processes of PUFAs involve enzymatic breakdown, emulsification, and uptake by specialized transport proteins in the small intestine, ensuring their distribution to tissues for utilization [39]. The digestibility of PUFAs, like other lipids, decreases with increasing chain length and is influenced by factors such as dietary composition and individual biological differences [40]. Furthermore, the synergistic effects of specific nutrients, including antioxidants such as vitamin E, vitamin D, and vitamin B6, have the potential to enhance and amplify the beneficial effects of PUFAs [41,42,43].

Our research has several notable advantages. Firstly, we utilized a substantial number of 8842 participants, and we considered weight, which ensured that our findings reflect American adults. Secondly, this is the first investigation into the combined impacts of three types of fatty acids on low muscle mass. Finally, we employed various statistical methodologies to comprehensively examine the connection between fatty acids and low muscle mass. This multifaceted method enhances the trustworthiness and robustness of our findings.

Several limitations must be acknowledged. First, the intake of fatty acids in our study was based on dietary recalls, which may introduce recall bias. Second, we have considered possible confounders, like demographic characteristics, lifestyle, habits, and medical history. However, there may still be other unidentified confounding factors that have not been adequately addressed, such as environmental factors and gut microbiota. Furthermore, this study was constrained by the NHANES data, and future research is needed to diversify populations. Last, as this is a cross-sectional study, longitudinal studies are needed to clarify the causal relationship and the long-term effects of PUFA on low-muscle-mass risk. Considering these limitations, it is clear that further research is required to address these issues and strengthen our understanding of dietary fatty acids in health. Nonetheless, our study identified a correlation between dietary fatty acids and low muscle mass, perhaps offering evidence for clinical application.

## 5. Conclusions

Our findings revealed an inverse association between three combined types of fatty acids and low-muscle-mass prevalence, with PUFAs being the primary contributors. Specifically, LA and ALA were significantly linked to a reduced risk of low muscle mass among PUFAs. Additionally, an interaction between PUFAs and obesity was observed, suggesting a stronger relationship between PUFA intake and low muscle mass in obese individuals.

## Figures and Tables

**Table 1:** Baseline characteristics.

	All	Non-Sarcopenia	Sarcopenia	*p* Value
	N = 8842	N = 8135	N = 707	
Age, (years)	37.0 [27.0; 48.0]	37.0 [27.0; 48.0]	45.0 [32.0; 53.0]	<0.001
Gender, n (%)				0.737
Female	4437 (50.2%)	4087 (50.2%)	350 (49.5%)	
Male	4405 (49.8%)	4048 (49.8%)	357 (50.5%)	
Ethnicity, n (%)				<0.001
Mexican American	1351 (15.3%)	1100 (13.5%)	251 (35.5%)	
Non-Hispanic Black people	1783 (20.2%)	1737 (21.4%)	46 (6.51%)	
Non-Hispanic White people	3270 (37.0%)	3075 (37.8%)	195 (27.6%)	
Other	2438 (27.6%)	2223 (27.3%)	215 (30.4%)	
Household economic level, n (%)				<0.001
High	2761 (31.2%)	2609 (32.1%)	152 (21.5%)	
Low	2893 (32.7%)	2596 (31.9%)	297 (42.0%)	
Median	3188 (36.1%)	2930 (36.0%)	258 (36.5%)	
Educational attainment, n (%)				<0.001
Higher or equal	7328 (82.9%)	6826 (83.9%)	502 (71.0%)	
Lower than high school	1514 (17.1%)	1309 (16.1%)	205 (29.0%)	
PUFA (gm)	17.5 [11.2; 25.8]	17.6 [11.4; 26.1]	15.1 [9.59; 22.1]	<0.001
PUFA Quartiles (gm)				<0.001
Q1 (≤11.2)	2207 (25.0%)	1974 (24.3%)	233 (33.0%)	
Q2 (11.2–17.5]	2231 (25.2%)	2045 (25.1%)	186 (26.3%)	
Q3 (17.5–25.8]	2195 (24.8%)	2021 (24.8%)	174 (24.6%)	
Q4 (>25.8)	2209 (25.0%)	2095 (25.8%)	114 (16.1%)	
MUFA (gm)	26.8 [17.8; 38.4]	27.2 [18.1; 38.7]	23.3 [15.9; 33.9]	<0.001
MUFA Quartiles (gm)				<0.001
Q1 (≤17.8)	2212 (25.0%)	1989 (24.4%)	223 (31.5%)	
Q2 (17.8–26.8]	2200 (24.9%)	1998 (24.6%)	202 (28.6%)	
Q3 (26.8–38.4]	2217 (25.1%)	2064 (25.4%)	153 (21.6%)	
Q4 (>38.4)	2213 (25.0%)	2084 (25.6%)	129 (18.2%)	
SFA (gm)	24.2 [15.6; 36.1]	24.5 [15.8; 36.4]	21.4 [13.1; 33.4]	<0.001
SFA Quartiles (gm)				<0.001
Q1 (≤15.6)	2215 (25.1%)	1988 (24.4%)	227 (32.1%)	
Q2 (15.6–24.2]	2204 (24.9%)	2023 (24.9%)	181 (25.6%)	
Q3 (24.2–36.1]	2202 (24.9%)	2048 (25.2%)	154 (21.8%)	
Q4 (>36.1)	2221 (25.1%)	2076 (25.5%)	145 (20.5%)	
Smoking status, n (%)				0.252
No	5507 (62.3%)	5052 (62.1%)	455 (64.4%)	
Yes	3335 (37.7%)	3083 (37.9%)	252 (35.6%)	
Alcohol, n (%)				<0.001
No	1282 (14.5%)	1120 (13.8%)	162 (22.9%)	
Yes	7560 (85.5%)	7015 (86.2%)	545 (77.1%)	
BMI (kg/m^2^)	27.6 [23.7; 32.3]	27.1 [23.5; 31.6]	33.5 [29.0; 39.3]	<0.001
Obesity, n (%)				<0.001
No	5682 (64.3%)	5462 (67.1%)	220 (31.1%)	
Yes	3160 (35.7%)	2673 (32.9%)	487 (68.9%)	
Sedentary behavior, n (%)				0.035
<4 h	2051 (23.2%)	1866 (22.9%)	185 (26.2%)	
4–6 h	2083 (23.6%)	1906 (23.4%)	177 (25.0%)	
6–8 h	2587 (29.3%)	2384 (29.3%)	203 (28.7%)	
>8 h	2121 (24.0%)	1979 (24.3%)	142 (20.1%)	
Diabetes mellitus, n (%)				<0.001
No	7913 (89.5%)	7358 (90.4%)	555 (78.5%)	
Yes	929 (10.5%)	777 (9.55%)	152 (21.5%)	
Hypertension, n (%)				<0.001
No	6498 (73.5%)	6065 (74.6%)	433 (61.2%)	
Yes	2344 (26.5%)	2070 (25.4%)	274 (38.8%)	

PUFA: polyunsaturated fatty acid; MUFA: monounsaturated fatty acid; SFA: saturated fatty acid. The frequency proportions showed categorical variables, while the mean and the first and third quartiles showed continuous variables.

**Table 2:** ORs (95% CIs) for sarcopenia in the fatty acid quartiles.

	Quartiles of the Fatty Acids (gm)				*p* for Trend
	Q1	Q2	Q3	Q4	
SFA	(≤15.6)	(15.6–24.2]	(24.2–36.1]	(>36.1)	
Crude	1	0.71 [0.55–0.93]	0.68 [0.52–0.89]	0.59 [0.41–0.84]	0.005
Model 1	1	0.74 [0.56–0.98]	0.71 [0.54–0.94]	0.57 [0.39–0.83]	0.006
Model 2	1	0.71 [0.54–0.94]	0.65 [0.47–0.89]	0.51 [0.35–0.76]	0.002
MUFA	(≤17.8)	(17.8–26.8]	(26.8–38.4]	(>38.4)	
Crude	1	0.92 [0.71–1.19]	0.66 [0.50–0.86]	0.59 [0.44–0.79]	<0.001
Model 1	1	0.92 [0.71–1.20]	0.65 [0.48–0.88]	0.55 [0.40–0.75]	<0.001
Model 2	1	0.90 [0.69–1.17]	0.64 [0.47–0.88]	0.52 [0.38–0.71]	<0.001
PUFA	(≤11.2)	(11.2–17.5]	(17.5–25.8]	(>25.8)	
Crude	1	0.75 [0.57–0.98]	0.72 [0.57–0.91]	0.48 [0.35–0.66]	<0.001
Model 1	1	0.76 [0.57–1.01]	0.70 [0.55–0.90]	0.48 [0.34–0.67]	<0.001
Model 2	1	0.78 [0.58–1.06]	0.71 [0.54–0.93]	0.48 [0.34–0.69]	<0.001

Q: quartile. Crude: non-adjusted. Model 1: adjusted for age, gender, ethnicity, household economic level, and educational attainment. Model 2: adjusted for age, gender, ethnicity, household economic level, educational attainment, smoking status, drinking status, diabetes mellitus, hypertension, and obesity.

**Table 3:** Weighted quantile sum regression analysis.

	Estimate	Standard Error	Z Value	*p* Value
Intercept	−2.09836	0.07787	−26.946	<0.001
WQS	−0.21840	0.04636	−4.711	<0.001

**Table 4:** Mediation effect.

Pathways	Mediation Proportions	95%CI	*p*-Value
PUFAs→Albumin→Low muscle mass	2.64%	5.60 × 10^−3^, 0.06	0.03
PUFAs→White blood cell→Low muscle mass	3.75%	0.02, 0.06	<0.001
PUFAs→Bilirubin→Low muscle mass	1.60%	6.42 × 10^−4^, 0.04	0.042
PUFAs→Gamma-glutamyltransferase→Low muscle mass	1.14%	4.12 × 10^−3^, 0.02	0.004

**Table 5:** Association of types of polyunsaturated fatty acids with sarcopenia.

	OR [95%CI]/*p* Value	
PUFAs (gm)	Unadjusted	Adjusted Model
C18:3		
Per SD increment	0.75 [0.68–0.82]/<0.001	0.73 [0.66–0.81]/<0.001
T1	1	1
T2	0.91 [0.69–1.19]	0.90 [0.67–1.19]
T3	0.65 [0.48–0.88]	0.63 [0.45–0.87]
*p* value for trend	<0.001	<0.001
C20:5		
Per SD increment	0.97 [0.88–1.05]/0.48	0.98 [0.88–1.07]/0.66
T1	1	1
T2	0.97 [0.76–1.24]	0.94 [0.73–1.21]
T3	0.76 [0.59–0.97]	0.72 [0.55–0.95]
*p* value for trend	0.41	0.33
C22:5		
Per SD increment	0.95 [0.85–1.03]/0.30	0.92 [0.80–1.02]/0.15
T1	1	1
T2	1.02 [0.77–1.35]	0.89 [0.66–1.20]
T3	0.97 [0.73–1.30]	0.81 [0.59–1.11]
*p* value for trend	0.33	0.08
C22:6		
Per SD increment	0.96 [0.86–1.04]/0.39	0.93 [0.82–1.03]/0.20
T1	1	1
T2	0.96 [0.76–1.21]	0.95 [0.75–1.22]
T3	0.77 [0.60–1.00]	0.68 [0.52–0.88]
*p* value for trend	0.24	0.11
C18:2		
Per SD increment	0.71 [0.64–0.78]/<0.001	0.71 [0.64–0.79]/<0.001
T1	1	1
T2	0.76 [0.59–0.97]	0.77 [0.59–0.99]
T3	0.54 [0.42–0.72]	0.52 [0.38–0.72]
*p* value for trend	<0.001	<0.001
C20:4		
Per SD increment	0.93 [0.85–1.00]/0.06	0.85 [0.78–0.94]/<0.001
T1	1	1
T2	0.99 [0.78–1.27]	0.88 [0.67–1.15]
T3	0.97 [0.76–1.23]	0.75 [0.57–1.00]
*p* value for trend	0.44	0.03

C18:2 (linoleic acid), C18:3 (alpha-linolenic acid), C20:4 (arachidonic acid), C20:5 (eicosa-pentaenoic acid), C22:5 (docosapentaenoic acid), and C22:6 (docosa-hexaenoic acid). Adjusted model: adjusted for age, gender, ethnicity, household economic level, educational attainment, smoking status, alcohol, diabetes mellitus, hypertension, and obesity.

## Data Availability

The data used for these analyses are all publicly available online (https://wwwn.cdc.gov/nchs/nhanes/Default.aspx) (accessed on 23 February 2025).

## Abbreviations

The following abbreviations are used in this manuscript:

PUFA	polyunsaturated fatty acid
MUFA	monounsaturated fatty acid
SFA	saturated fatty acid
NHANES	National Health and Nutrition Examination Survey
RCS	restricted cubic splines
WQS	weighted quantile sum
LA	linoleic acid
ALA	alpha-linolenic acid
EPA	eicosa-pentaenoic acid
DHA	docosa-hexaenoic acid
DXA	dual-energy X-ray absorptiometry
MEC	mobile examination center
PIR	poverty income ratio
BMI	body mass index
LMM	low muscle mass
